# Supplementary material for: PPa1 insufficiency drives lysosomal storage disease and inflammatory macrophage expansion in the bone marrow
Source: bioRxiv. 2026 Mar 18:2026.03.16.712247. Preprint. [Version 1] doi: 10.64898/2026.03.16.712247 (PMC13015360; doi:10.64898/2026.03.16.712247)
Supplement: 4 [file NIHPP2026.03.16.712247v1-supplement-4.pdf]

bioRxiv preprint doi: https://doi.org/10.64898/2026.03.16.712247; this version posted March 18, 2026. The copyright holder for this preprint (which was not certified by peer review) is the author/funder, who has granted bioRxiv a license to display the preprint in perpetuity. It is made available under aCC-BY 4.0 International license.

FIGURE S1

a

Offspring from Ppa1$^{+/hpt}$ X Ppa1$^{+/-}$

Genotype	Observed	Expected
Ppa1$^{+/+}$	319 (42.3%)	188.75 (25%)
Ppa1$^{hpt/+}$	158 (20.9%)	188.75 (25%)
Ppa1$^{+/-}$	253 (33.5%)	188.75 (25%)
Ppa1$^{hpt/-}$	25 (3.3%)	188.75 (25%)
Total	755	755

Offspring from Ppa1$^{+/hpt}$ X Ppa1$^{+/hpt}$

Genotype	Observed	Expected
Ppa1$^{+/+}$	68 (30.1%)	56.5 (25%)
Ppa1$^{hpt/+}$	112 (49.5%)	113 (50%)
Ppa1$^{hpt/hpt}$	46 (20.3%)	56.5 (25%)
Total	226	226

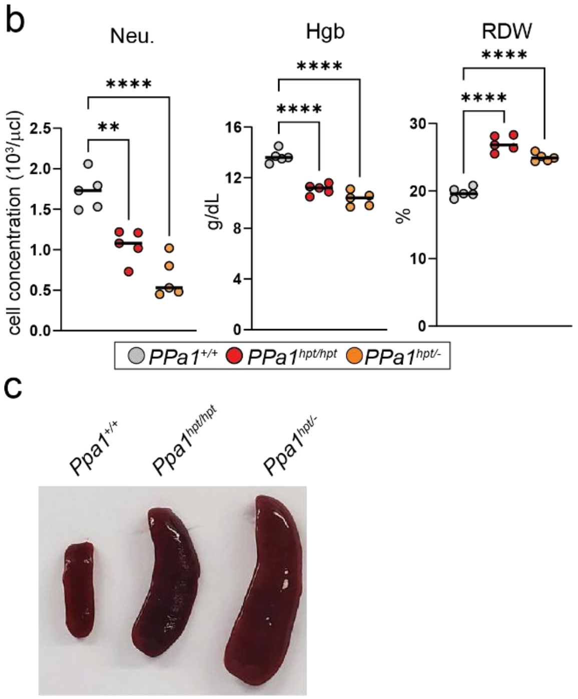

Figure S1: Comparison of *PPa1$^{hpt/-}$* and *PPa1$^{hpt/hpt}$* mouse strains. (a) Observed Mendelian inheritance ratios of *PPa1$^{hpt/-}$* and *PPa1$^{hpt/hpt}$* mice from the indicated breeding crosses. **(b)** Peripheral blood neutrophil counts and RBC indices in *PPa1$^{hpt/-}$* and *PPa1$^{hpt/hpt}$* mice. Each symbol represents an individual mouse. Horizontal bars represent the median and statistical significance was determined with one-way ANOVA with Dunnett's multiple comparisons. **(c)** Representative splenomegaly in *PPa1$^{hpt/-}$* and *PPa1$^{hpt/hpt}$* mice.

bioRxiv preprint doi: https://doi.org/10.64898/2026.03.16.712247; this version posted March 18, 2026. The copyright holder for this preprint (which was not certified by peer review) is the author/funder, who has granted bioRxiv a license to display the preprint in perpetuity. It is made available under aCC-BY 4.0 International license.

FIGURE S2

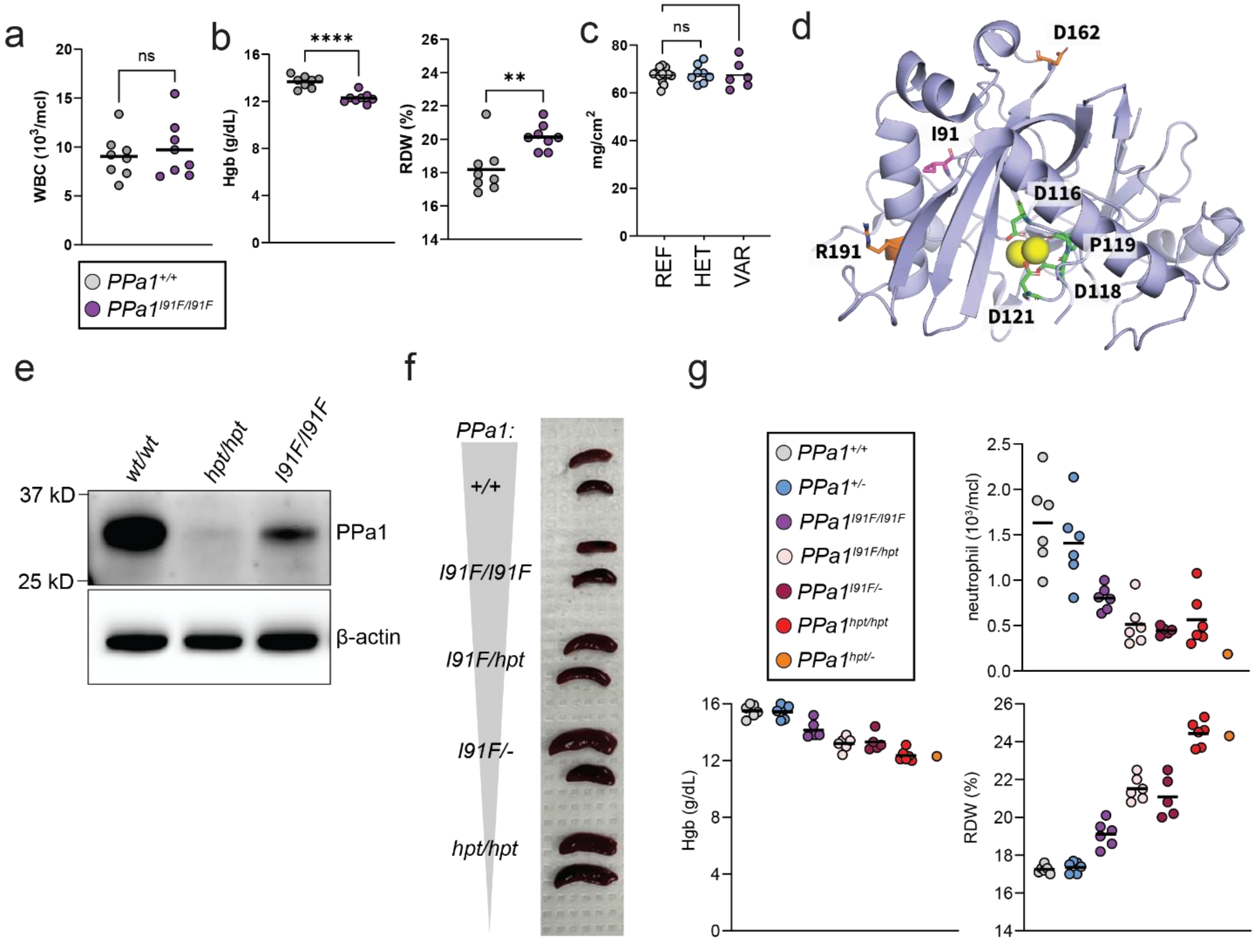

Figure S2: Characterization of PPa1$^{I91F/I91F}$ mutant mice. (a) Peripheral WBC counts in *PPa1$^{I91F/I91F}$* mice. **(b)** RBC indices in *PPa1$^{I91F/I91F}$* mice. **(c)** Tibial bone mineral density in *PPa1$^{I91F/I91F}$* mice. **(d)** Structural localization of I91 (purple) within the crystal structure of human PPA1, shown relative to the active site (green) and R191 and D162 (orange). **(e)** Immunoblot analysis of PPa1 expression in bone marrow from the indicated genotypes. **(f)** Spleen size as it relates to *PPa1* gene dosing. **(g)** Peripheral neutrophil counts and RBC indices across an allelic series of PPa1 variants. Each symbol represents an individual mouse. Horizontal bars represent the mean and statistical significance was determined with two-tailed unpaired t-test **(a, b)** and one-way ANOVA with Dunnett's multiple comparisons **(c)**.

bioRxiv preprint doi: https://doi.org/10.64898/2026.03.16.712247; this version posted March 18, 2026. The copyright holder for this preprint (which was not certified by peer review) is the author/funder, who has granted bioRxiv a license to display the preprint in perpetuity. It is made available under aCC-BY 4.0 International license.

FIGURE S3

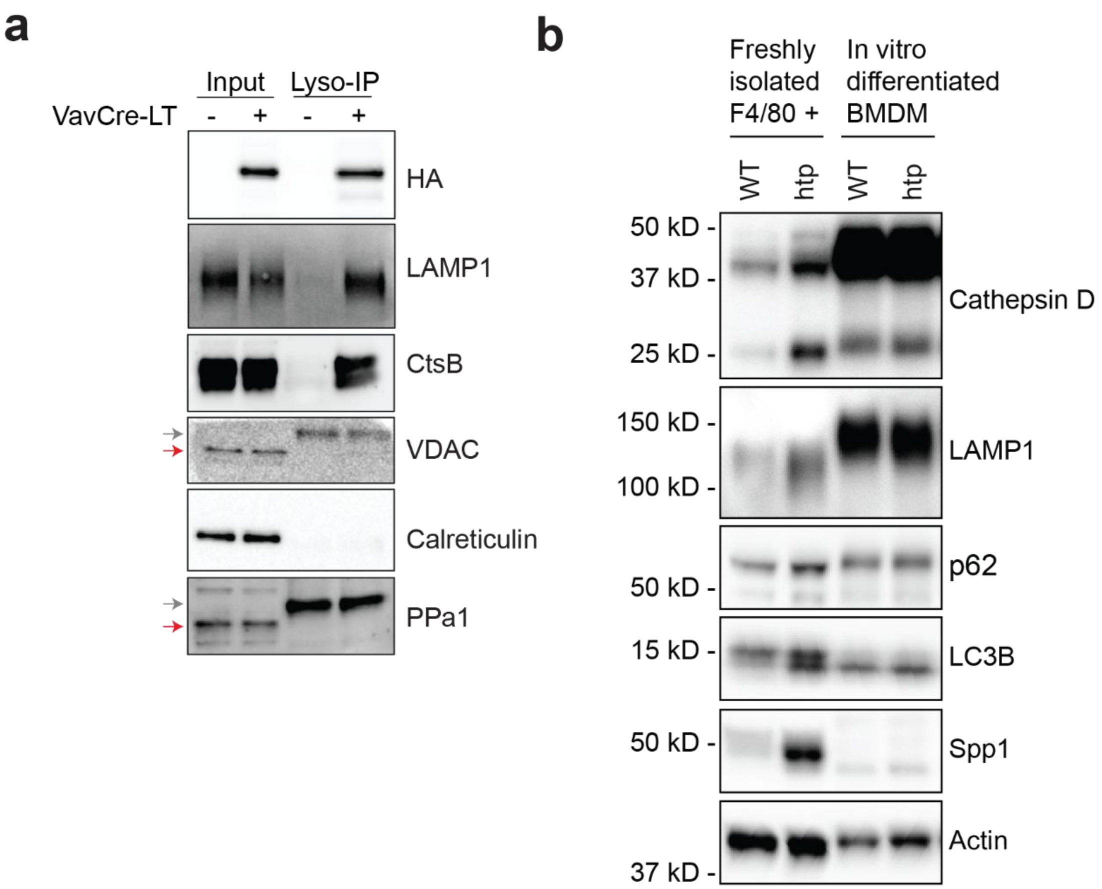

Figure S3: Lysosomal markers in PPa1-deficient cells. (a) Lyso-IP from mice expressing 3xHA-TMEM192 within their hematopoietic cells showing that PPa1 does not appear in the lysosomal fraction. Red arrows indicate expected band, gray arrows indicate background band. Data is representative of two independent Lyso-IP experiments. **(b)** Comparison of lysosomal markers in freshly isolated F4/80+ bone marrow macrophages and BMDMs differentiated in vitro for 6 days with CSF-1. Data is representative of three independent BMDM culture experiments.
